# Alignment of natural language processing-derived RDoC scores with depression, anhedonia, and anxiety in psychiatric electronic health records

**DOI:** 10.1038/s44277-026-00070-3

**Published:** 2026-09-30

**Authors:** Manuel Kuhn, Seoin Ahn, Vincent Holstein, Annah Rossvall, Savannah Layfield, Lucie A. Duffy, Samantha Wong, Robyn Ellis, Eliot Gelwan, Fernando Rodriguez-Villa, Agustin Yip, Ipsit Vahia, Diego A. Pizzagalli, Kerry J. Ressler, Jonathan Vogelgsang

**Affiliations:** 1 McLean Hospital, Mass General Brigham Department of Psychiatry, Harvard Medical School, Belmont, MA USA; 2 Institute of Medical Psychology, Charité - Universitätsmedizin Berlin, Corporate member of Freie Universität Berlin and Humboldt-Universität zu Berlin, Berlin, Germany; 3 Noel Drury, M.D. Institute for Translational Depression Discoveries, University of California, Irvine, CA USA

**Keywords:** Depression, Anxiety

## Abstract

Traditional categorical diagnoses often fail to capture the dimensional nature of psychiatric disorders such as depression and anxiety. Natural Language Processing (NLP) offers a promising approach to extract dimensional constructs relevant to psychopathology from clinical documentation. In this cross-sectional study, we analyzed clinical notes from 358 psychiatric patients treated at McLean Hospital and enrolled in the “Psychometrics at McLean” (PAM) study to examine the alignment between NLP-derived Research Domain Criteria (RDoC) constructs and psychometric assessments of depression, anhedonia, and anxiety. RDoC scores for Positive Valence Systems (PVS) and Negative Valence Systems (NVS) were extracted using NLP methods previously validated at the group level and compared with standardized clinical assessments: Positive Valence Systems Scale (PVSS), Quick Inventory of Depressive Symptomatology (QIDS), Snaith-Hamilton Pleasure Scale (SHAPS), and Generalized Anxiety Disorder-7 (GAD-7). Significant associations were observed between depression (QIDS) and both PVS (β = −0.42, *p* = 0.008) and NVS (β = 0.45, *p* = 0.015); anhedonia (SHAPS) and both PVS (β = −0.39, *p* = 0.005) and NVS (β = 0.36, *p* = 0.030); and anxiety (GAD-7) and NVS (β = 0.29, *p* = 0.049), but not anxiety and PVS. These results are consistent with theoretical accounts in which both PVS and NVS are implicated in depression, whereas anxiety primarily involves NVS. These findings demonstrate that NLP-derived RDoC scores align with established clinical measures, providing evidence of convergent validity with symptom-based measures and of discriminant validity, most notably the selective association of anxiety with NVS. NLP-derived RDoC scores may offer a scalable approach for characterizing dimensional psychopathology; however, longitudinal and predictive validation will be required before applications such as treatment-response monitoring can be supported.

## Lay Summary

Psychiatric symptoms can be difficult to measure from routine clinical records. We used natural language processing to identify symptom dimensions from clinical notes and compared these measures with standardized questionnaires completed by psychiatric patients. The results showed meaningful relationships between language-derived measures and symptoms of depression, anhedonia, and anxiety. These findings suggest that clinical notes may provide useful information about dimensional psychiatric symptoms and could support scalable approaches to studying mental health.

## Introduction

Major depressive disorder (MDD) and anxiety disorders are highly prevalent, affecting hundreds of millions of individuals globally, and together represent leading causes of disability worldwide [1, 2]. These disorders are associated with substantial personal suffering, impaired functioning, and increased risk of chronic medical conditions as well as premature mortality [1, 3]. Despite their public health impact, traditional categorical diagnoses in psychiatry, as defined in the current Diagnostic and Statistical Manual of Mental Disorders (DSM) and International Classification of Diseases (ICD) classification systems, often lack sensitivity to the heterogeneity and dimensional nature of symptom presentations, which limits progress in identifying underlying mechanisms and developing personalized interventions [4, 5].

To address these challenges, the National Institute of Mental Health introduced the Research Domain Criteria (RDoC) framework [6], which promotes a dimensional and transdiagnostic research approach to psychopathology. Rather than focusing on traditional diagnostic boundaries, RDoC conceptualizes functioning across core domains, including Negative and Positive Valence Systems, Cognitive Systems, Social Processes, Arousal and Regulatory Systems, and Sensorimotor Systems, which are examined across multiple units of analysis (e.g., genes, neural circuits, behavior, self-report) and can range from adaptive to pathological variation [4, 5].

Among the six RDoC domains, the Positive Valence Systems (PVS) and Negative Valence Systems (NVS) are particularly relevant for depression and anxiety. While other constructs such as cognitive control, social processes, and arousal regulation have been implicated in psychopathology, impairments in reward-related processes, as reflected in the PVS, and heightened sensitivity to threat and aversion, as reflected in NVS, represent central features of mood and anxiety disorders [7]. In detail, PVS encompasses reward responsiveness, reward learning, and reward valuation, which are often diminished in depression and manifest clinically as anhedonia and reduced goal-directed behavior [8, 9]. In contrast, NVS refers to acute and sustained responses to aversive contexts, including acute threat (fear), potential threat (anxiety), and sustained threat, and is consistently elevated in both depression and anxiety disorders [10]. As such, an RDoC-informed dimensional approach makes it possible to capture dimensions of dysfunction that may be overlooked when individuals are grouped solely by traditional categorical diagnoses, thereby providing a more precise characterization of symptom profiles and their clinical relevance. This is particularly applicable in depression and anxiety, where overlapping features such as anhedonia, hypervigilance, anxiety, and altered stress reactivity can manifest at different levels of severity across individuals [7]. Thus, leveraging RDoC-derived measures of PVS and NVS has the potential to facilitate the identification of transdiagnostic markers that better explain individual differences in risk, course, and treatment response.

Recent advances in computational approaches, introducing precision medicine into psychiatry [11–13], particularly Natural Language Processing (NLP), have enabled the extraction of meaningful clinical information from Electronic Health Records (EHRs), providing a new avenue for high-throughput psychiatric phenotyping. Specifically, previous work by McCoy and colleagues demonstrated the feasibility of using NLP to derive RDoC-based symptom dimensions from psychiatric clinical notes [14]. However, further validation is necessary to establish the clinical utility of these extracted scores. The dimensional phenotyping approach used here (the publicly available CQH-Dimensional-Phenotyper) has been applied across multiple studies using large EHR cohorts, with its implementation publicly released, in which NLP-derived RDoC dimensions were associated with clinically meaningful outcomes including risk for suicide and accidental death [15], dementia risk [16], psychiatric emergency service outcomes [17], and COVID-19 hospitalization outcomes [18]. Notably, these prior applications have operated largely at the group or population level, relating aggregate NLP-derived scores to distal clinical outcomes. To our knowledge, these dimensions have not previously been validated at the level of the individual patient against concurrently collected, standardized psychometric measures of depression, anhedonia, and anxiety in an inpatient psychiatric cohort. The present study therefore extends this prior work by shifting the unit of analysis from group-level outcome associations to individual-level validation, evaluating the convergent and discriminant validity of NLP-derived RDoC scores against standardized self-report measures collected on the same day as psychiatric clinical documentation, and clarifying which symptom dimensions these scores do and do not track.

Here, we examined the alignment between NLP-derived RDoC scores and validated psychometric measures of depression, anhedonia, and anxiety in a well-characterized clinical cohort. In particular, we aimed to establish whether these computationally generated scores show convergent validity with established symptom measures and discriminant validity across symptom dimensions, as an initial step toward their potential use as scalable tools for psychiatric research.

## Materials and methods

### Dataset

8739 aggregated daily clinical notes from a trans-diagnostic sample comprising 358 psychiatric inpatients treated at McLean Hospital (Belmont, MA, USA) were used. Patients were enrolled in the “Psychometrics at McLean” (PAM) Project, a naturalistic study monitoring patients with mood and anxiety disorders who received standard clinical care plus additional psychometric evaluations (see Table 1 for clinical diagnoses, demographic information and clinical measures).

**Table 1:** Baseline characteristics of the study participants (*N* = 358).

Variable		n	Mean	STD
Participants		358		
Age (years)		323	33.8	13
Sex at birth (%)	Male	137 (38.3%)		
	Female	221 (61.7%)		
PVSS (Positive Valence)		323	91	28.3
QIDS (Depression)		101	15.5	5.7
SHAPS (Anhedonia)		127	4.8	4.1
GAD-7 (Anxiety)		153	14.2	5.6

Summary statistics are based on the participants included in each analysis. Sample sizes differ because of questionnaire availability and complete-case analysis.

Clinical documentation was accessed through the Electronic Data Warehouse (EDW) via ERISOne Jupyter Notebook on a hospital-owned, PHI-compliant server. Concatenated files were created from all medical records authored by psychiatrists, nurses, group therapists, and mental health specialists on the day of psychometric evaluation. These records included information on psychiatric symptoms, behavioral observations, and medication use. This comprehensive approach ensured that the analysis captured the full spectrum of clinical observations documented by the multidisciplinary treatment team. Patients were receiving inpatient psychiatric care at the time of psychometric evaluation. The notes analyzed therefore represent routine clinical documentation generated in the course of care on the assessment day, rather than standardized research intake assessments; this feature is considered further in the Discussion.

All participants provided written informed consent, and all study procedures were approved by the Mass General Brigham Human Research Committee, which serves as the Institutional Review Board for McLean Hospital.

### Natural language processing and RDoC score extraction

NLP-derived RDoC scores were obtained using a method developed by McCoy and colleagues [14] and previously validated at the group level. This method uses a dictionary-based approach and topic modeling techniques to extract symptom dimensions from unstructured clinical text. The NLP model used in this study identifies five RDoC domains (PVS, NVS, cognitive, arousal, and social processes) but does not include the sensorimotor systems domain which was added to the RDoC system on January 14, 2019. The algorithm applies a lexical analysis approach based on curated dictionaries from prior work, quantifying the frequency of RDoC-related terms in clinical text. In addition, topic modeling is used to identify patterns of co-occurring terms and underlying symptom dimensions, which inform dimensional scores for each RDoC domain. Text normalization and tokenization techniques were applied to ensure consistency in term identification. The publicly available implementation of this method was used [19]. Importantly, the pipeline performs lexical matching without negation detection, and therefore does not distinguish asserted symptoms (e.g., “severe anxiety”) from negated or ruled-out mentions (e.g., “no history of anxiety”); the implications of this property for interpretation are addressed in the Discussion.

For each patient, we created concatenated files of all clinical documentation by psychiatrists, nurses, group therapists, and mental health specialists on the day of psychometric evaluation to ensure comprehensive capture of clinical observations. This NLP-derived RDoC approach has previously been described and applied to characterize dimensional psychopathology and examine clinically meaningful outcomes in psychiatric populations [14–18].

### Psychometric measures

Symptom severity was assessed using the following validated psychometric instruments as part of the PAM study protocol: depression via the 16-item Quick Inventory of Depressive Symptomatology (QIDS; Cronbach’s α = 0.86) [20], anhedonia via the 14-item Snaith-Hamilton Pleasure Scale (SHAPS; internal consistency = 0.86) [21], anxiety via the 7-item Generalized Anxiety Disorder-7 (GAD-7; Cronbach’s α = 0.92) [22], and positive valence via the 21-item Positive Valence Systems Scale (PVSS; Cronbach’s α ≥ 0.91) [23].

### Statistical analysis

All analyses were conducted using R (version 4.1.0; R Foundation for Statistical Computing [24]). The ‘psych' [25] package was used for correlation analyses, while ‘lm’ function from base R was employed for multiple regression models. To assess potential multicollinearity between RDoC domains, Variance Inflation Factors (VIFs) were calculated using the ‘car’ package in R. We used a threshold of VIF < 5 to indicate acceptable levels of multicollinearity. Pearson correlation coefficients were computed to examine bivariate relationships between RDoC domains and psychometric measures. Statistical significance was set at p < 0.05 for all analyses.

For each psychometric measure (QIDS, SHAPS, GAD-7, and PVSS), separate multiple linear regression models were constructed with the five RDoC domains (PVS, NVS, cognitive, arousal, and social processes) as predictors, controlling for age and sex. Regression analyses were conducted using complete-case analysis. Consequently, the analytic sample for each questionnaire could be smaller than the number of participants who completed the questionnaire because participants with missing covariate data were excluded.

This approach allowed us to assess the independent contribution of each of the included RDoC domains while controlling for the influence of other domains and demographic factors. Psychometric assessment was available for: PVSS (n = 323), QIDS (n = 101), SHAPS (n = 127), and GAD-7 (n = 153). Because psychometric questionnaires were completed by different subsets of participants, we examined whether patients with and without a given questionnaire differed systematically. For each instrument, NLP-derived RDoC domain scores and demographic characteristics were compared between patients who did and did not complete that instrument (Supplementary Fig. 1, 2). PVSS was excluded from these comparisons because all participants completed the questionnaire; the smaller regression sample for PVSS resulted solely from missing covariate data under the complete-case analysis. In addition, to determine the extent to which the RDoC domain lexicons share lexical content—a possible source of inter-domain correlation under a string-matching method—we quantified the overlap in dictionary terms across domains using pairwise Jaccard similarity coefficients (Supplementary Fig. 3).

## Results

### Correlation patterns between RDoC domains and clinical measures

As shown in Table 2, VIF values for age and sex were consistently low (all VIFs < 1.10), indicating minimal collinearity. Among RDoC domains, arousal regulatory and cognitive domains showed moderate to high multicollinearity (VIFs ranging from 3.46–4.63), while NVS, PVS, and social domains exhibited moderate multicollinearity (VIFs between 2.11 and 3.68). Despite these elevated VIF values, we retained all predictors in our models for several reasons: (1) to maintain consistency with the RDoC framework that conceptualizes them as distinct but related constructs, (2) to preserve interpretability of domain-specific associations with clinical measures, and (3) the VIF values, while elevated, remained below the commonly used threshold of 5 for severe multicollinearity that would necessitate model adjustment.

**Table 2:** Variance Inflation Factors (VIF) for Predictors Across Regression Models.

Predictor	QIDS Model	SHAPS Model	GAD-7 Model	PVSS Model
Age	1.03	1.02	1.01	1.04
Sex at birth	1.09	1.08	1.07	1.03
Cognitive	4.03	3.77	3.56	3.46
Positive Valence	2.71	2.53	2.35	2.11
Negative Valence	3.68	3.63	3.24	3.00
Arousal Regulatory	4.57	4.63	4.25	4.10
Social	2.46	2.38	2.29	2.40

**Fig. 1:**
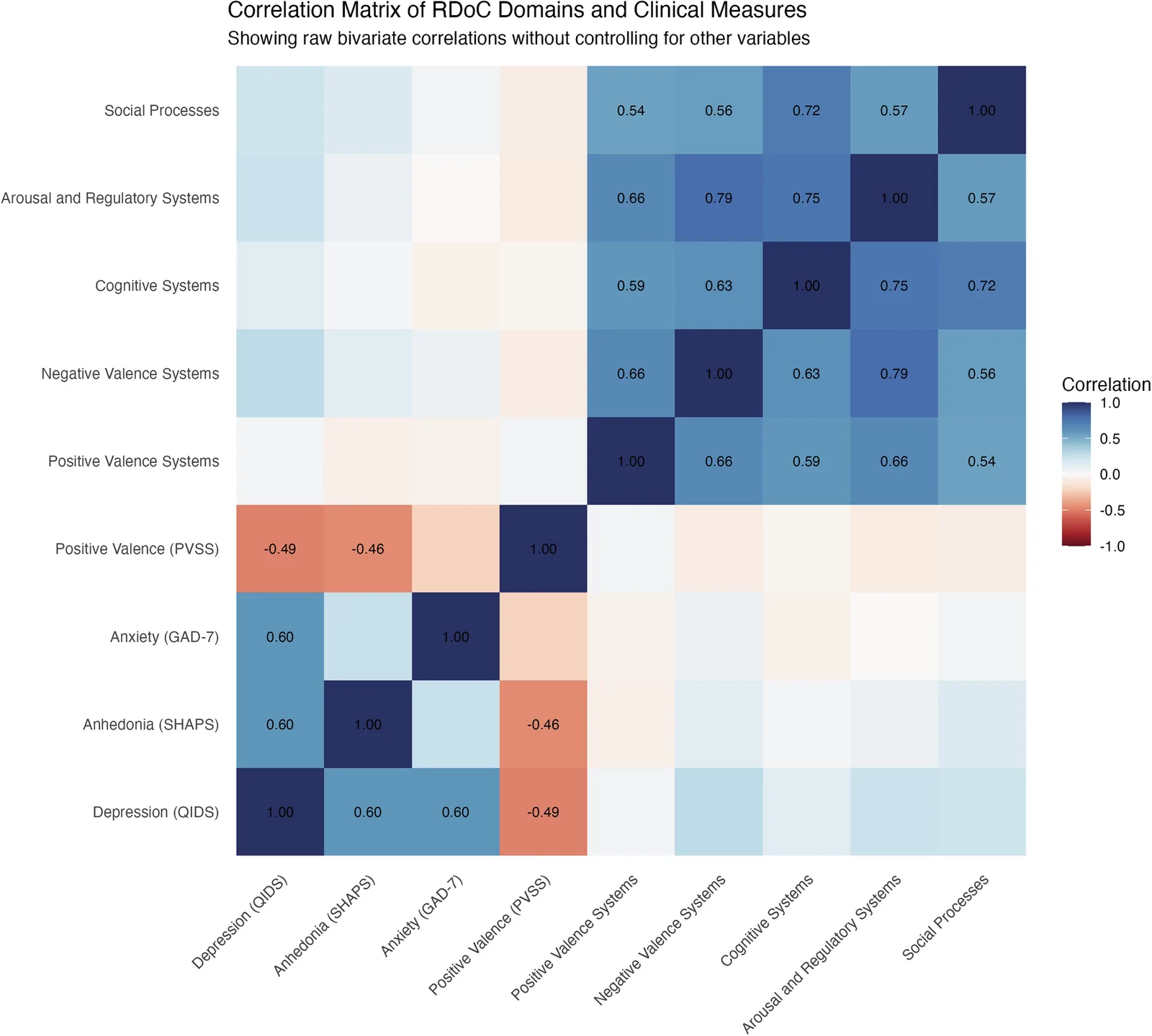
Bivariate correlation matrix of RDoC domains and clinical measures. Cells containing R-values are significantly correlated.

To understand the relationships between RDoC domains and psychometric measures, we first examined their bivariate correlations. As shown in Fig. 1, RDoC domains showed substantial intercorrelations (r = 0.54–0.79). Because the domain lexicons share little lexical content (pairwise Jaccard indices ranging from 0.006–0.184; Supplementary Fig. 3), the observed intercorrelations are unlikely to be explained solely by direct lexical overlap between dictionaries and are more consistent with co-occurring symptoms and shared documentation patterns across domains. Particularly robust relationships were observed between cognitive and arousal domains (r = 0.75), cognitive and social domains (r = 0.72), and NVS and arousal domains (r = 0.79). These correlations suggest that the NLP algorithm captures overlapping aspects of psychopathology across domains, reflecting the inherent complexity of psychiatric symptoms.

The correlation matrix (Fig. 1) also revealed expected relationships between clinical measures: depression (QIDS) and anhedonia (SHAPS) measures were strongly correlated (r = 0.60) and both were negatively associated with positive valence assessments (PVSS; r = −0.49 and −0.46, respectively). In contrast, the bivariate correlations between RDoC domains and clinical measures were more modest. To assess the independent contribution of each domain, we therefore fitted multiple regression models controlling for the remaining domains and for demographic covariates.

### Multiple regression analysis

**Fig. 2:**
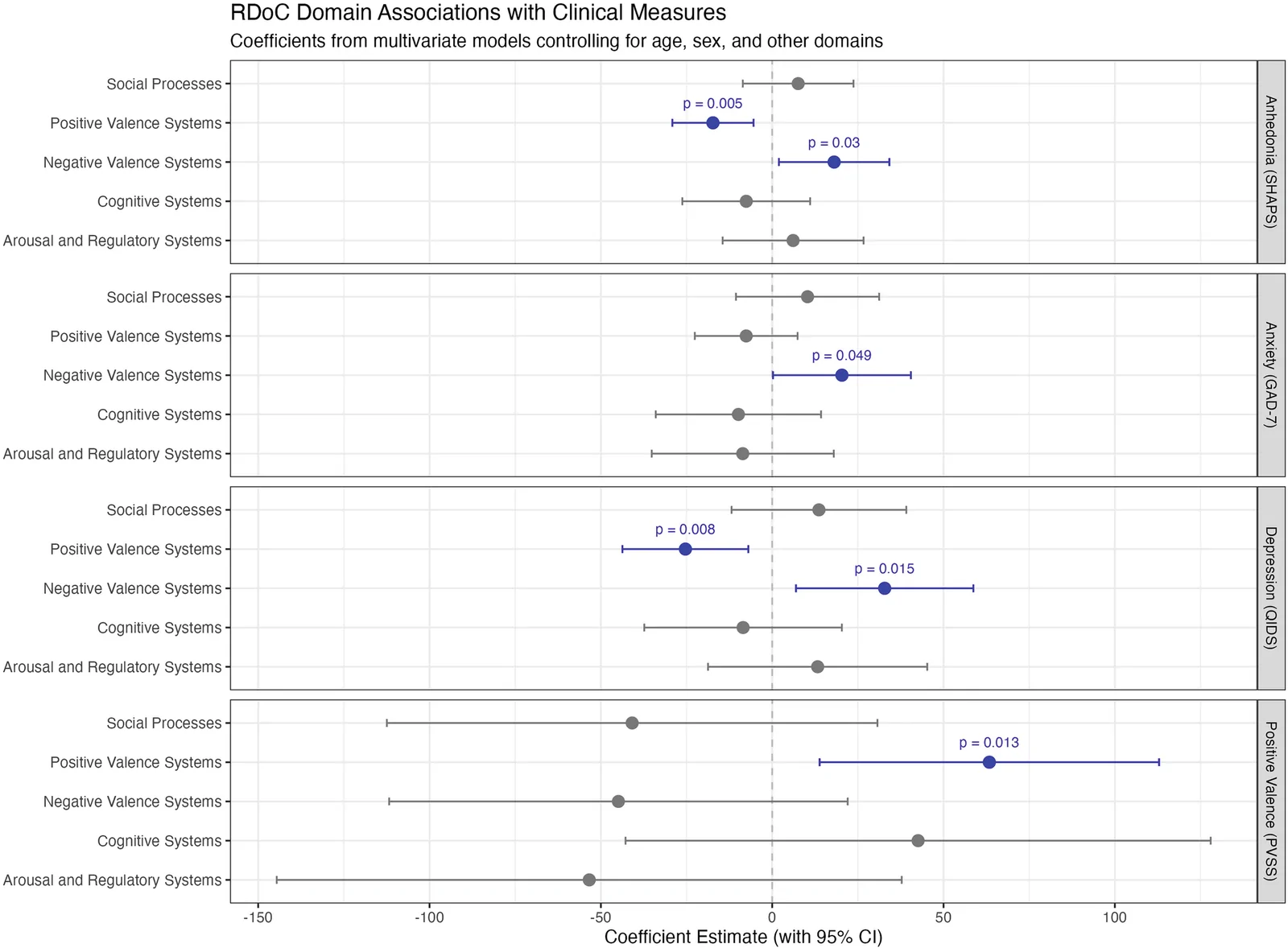
RDoC Domain associations with Clinical measures (SHAPS, GAD-7, QIDS, PVSS). Coefficients from multiple regression model controlling for age, sex, and other domains. Model R² ranged from 0.04 (PVSS)–0.18 (QIDS).

Figure 2 illustrates the results from our multiple regression models, showing the associations between each RDoC domain and psychometric measure after controlling for age, sex, and other RDoC domains **(**Table 3**)**. PVS showed significant negative associations with depression (β = −0.42, *p* = 0.008) and anhedonia (β = −0.39, *p* = 0.005), while NVS showed significant positive associations with depression (β = 0.45, *p* = 0.015), anhedonia (β = 0.36, *p* = 0.030), and anxiety (β = 0.29, *p* = 0.049). Additionally, PVS demonstrated a significant positive association with PVSS (β = 0.20, *p* = 0.013), providing convergent validity for our NLP-derived construct. The remaining RDoC domains did not show significant associations with any of the clinical measures after controlling for other variables. Several of these coefficients exceeded the corresponding bivariate correlations in magnitude (Fig. 1). Because the RDoC domains are substantially intercorrelated, entering them simultaneously removes variance shared across domains, allowing domain-specific associations to emerge, a pattern consistent with statistical suppression. We nevertheless interpret these coefficients cautiously, as suppression effects can be sensitive to sample size and to the specific set of covariates included.

**Table 3:** Significant Associations Between RDoC Domains and Psychometric Measures.

Psychometric Measure	RDoC Domain	β	*p*-value	Sample size
Depression (QIDS)	Positive Valence	−0.42	0.008^*^	101
	Negative Valence	0.45	0.015^*^	
Anhedonia (SHAPS)	Positive Valence	−0.39	0.005^*^	127
	Negative Valence	0.36	0.030^*^	
Anxiety (GAD-7)	Negative Valence	0.29	0.049^*^	153
Positive Valence Systems Scale (PVSS)	Positive Valence	0.20	0.013^*^	323

Asterisk denotes statistical significance at *p* < 0.05.

Despite the substantial intercorrelations between domains, the differential pattern of associations with clinical measures supports the discriminant validity of these domains. Specifically, the NVS was the only valence domain that showed a significant association with anxiety (GAD-7), while both PVS and NVS domains demonstrated distinct relationships with depression and anhedonia. Comparisons of patients with versus without each questionnaire revealed no consistent differences in demographic characteristics or NLP-derived RDoC domain scores across questionnaire-specific analytic samples (Supplementary Fig. 1), indicating that the subsamples used for each regression model were broadly comparable with respect to the measured variables. Multivariable analyses likewise identified no independent predictors of inclusion in questionnaire-specific analytic samples (Supplementary Fig. 2).

## Discussion

Our findings contribute to the growing body of literature supporting the importance of dimensional assessment of psychiatric symptoms using NLP. The observed associations between RDoC scores and psychometric measures support the convergent and discriminant validity of computationally derived symptom dimensions: positive and negative valence systems both tracked depression and anhedonia, whereas anxiety showed a selective negative-valence association. It is worth being precise about the nature of this validity. The instruments used here (QIDS, SHAPS, GAD-7, PVSS) are established symptom-severity measures rather than the transdiagnostic, construct-level instruments catalogued in the NIMH RDoC matrix. The present evidence therefore speaks to convergence with symptom severity and to discrimination across symptom dimensions, not to validation of the RDoC constructs themselves at the construct level. These patterns have several implications for research and clinical practice.

First, the alignment of both PVS and NVS with depression and anhedonia highlights the dual nature of MDD, characterized by both reward system deficits and heightened negative affectivity. The strong negative association between QIDS scores and positive valence alongside the positive association with negative valence supports a bidirectional relationship consistent with contemporary neurobiological models of depression that emphasize both reduced reward sensitivity and enhanced sensitivity to negative stimuli [26, 27]. This pattern parallels neuroimaging findings of reduced striatal activation to reward and increased amygdala reactivity to negative stimuli in depression [28]. Similarly, anhedonia shows a robust negative association with positive valence and a positive association with negative valence, supporting its conceptualization as a multidimensional construct involving both deficits in positive valence systems (reduced capacity for pleasure) and heightened negative valence systems (increased avoidance motivation). This dual pattern suggests that assessment of anhedonia may need to capture both valence systems rather than reward-related deficits alone; whether this distinction is informative for treatment selection remains to be tested.

Second, anxiety was associated selectively with NVS and, unlike depression and anhedonia, showed no significant association with PVS. This pattern suggests that anxiety, as measured here, is characterized by heightened negative valence without the accompanying reduction in positive valence observed for depression and anhedonia. The significant positive association between GAD-7 scores and negative valence, combined with the absence of any significant relationship with positive valence, suggests that anxiety is predominantly characterized by an overactive negative affect system. This distinction aligns with neuroimaging studies showing heightened activity in the amygdala and insula in response to threat-related stimuli in individuals with generalized anxiety disorder and other anxiety disorders [29, 30]. This finding has important treatment implications, suggesting that interventions specifically targeting the negative valence system—such as those addressing threat processing [31]—may be relevant to anxiety as well as to the negative-valence component of depression and anhedonia; the present cross-sectional data, however, do not permit conclusions about differential treatment efficacy across these presentations.

The correlation matrix between RDoC domains revealed substantial intercorrelations, particularly among cognitive, arousal, and social domains. These high correlations, alongside elevated VIF values, suggest some overlap in the constructs captured by the NLP algorithm. These findings suggest that while the NLP-derived RDoC domains share variance, they nevertheless capture clinically meaningful and distinct aspects of psychopathology. This interpretation is supported by the minimal lexical overlap between domain dictionaries (Supplementary Fig. 3), suggesting that the observed intercorrelations are unlikely to arise primarily from shared dictionary terms. Future refinement of the NLP algorithms could potentially improve discrimination between domains, perhaps by incorporating more context-aware natural language understanding techniques or by expanding the lexicon used to identify domain-specific terms. These findings extend a method whose prior validation has been established primarily at the group level. The publicly available dimensional phenotyper has been applied across multiple large EHR cohorts and associated with clinically meaningful outcomes, including suicide and accidental death, dementia onset, psychiatric emergency service outcomes, and COVID-19 hospitalization outcomes [15–18]. What has not been established is whether the same scores carry interpretable signal for an individual patient. By pairing each patient’s clinical documentation with standardized symptom measures administered on the same day, the present study addresses this gap directly. This constitutes a more stringent test than group-level outcome prediction, in which individual-level measurement error can average out across large samples: convergence with concurrent symptom severity, and discrimination across symptom dimensions, must hold patient by patient. Such individual-level evidence is a prerequisite for future patient-level research and potential clinical applications.

Several limitations of our study should be noted. First, our study was conducted at a single institution with a relatively homogeneous patient population, which may limit generalizability. Validation in diverse clinical settings and populations is necessary to establish the broad applicability of this approach. Second, medication use, comorbid psychiatric conditions, and time of evaluation in relation to the full hospital course were not included as covariates, as this study was an exploratory analysis designed to establish initial proof-of-concept relationships between NLP-derived measures and standard clinical assessments. Third, the analyzed language is clinician-derived rather than patient-derived. RDoC-related associations may therefore be shaped by documentation practices and by the more conventional diagnostic lens of the documenting clinicians and may not directly index patients’ subjective experience. This concern is compounded by the absence of negation detection in the NLP pipeline: because the method matches lexicon terms without distinguishing assertion from negation, a documented term may reflect an index of suspicion or the ruling out of an alternative diagnosis (e.g., “no evidence of mania”) rather than a present symptom. These properties are inherent to the established instrument and were out of scope to modify here, but they warrant caution in interpreting the magnitude and meaning of the observed associations. Fourth, the design is cross-sectional, and the four psychometric instruments were available for different and partially non-overlapping subsamples (n = 101–323). This limits direct comparison of effect sizes across models and precludes inference about the temporal stability or predictive utility of the NLP-derived scores. Although the subsample comparisons described above did not indicate systematic differences on the measured variables, residual selection effects cannot be excluded. Fifth, we observed substantial multicollinearity between RDoC domains, with variance inflation factors exceeding a value of 3 for cognitive, arousal, and negative domains. This indicates potential overlap in the constructs measured by these computational approaches and suggests that refinement of the NLP algorithm may be needed to better differentiate these domains. Sixth, while our multiple regression approach allowed us to control several factors, the modest R-squared values (ranging from 0.04–0.18, Supplementary Table 1) indicate that these models explain a relatively small proportion of the variance in psychometric scores. This is consistent with an exploratory study in a complex domain like psychopathology, but it does highlight the need for more comprehensive models that incorporate additional clinical variables and potentially biological markers.

In conclusion, this study demonstrates the feasibility of using NLP to extract clinically relevant RDoC scores from psychiatric EHRs. The observed associations between NLP-derived PVS/NVS scores and standardized psychometric assessments provide evidence of convergence with clinically assessed symptom severity. This approach may have potential applications in both clinical practice and research.

Future research should explore the longitudinal stability of these scores, their utility in predicting treatment response, and their integration with biological measures to advance our understanding of the neurobiological underpinnings of psychiatric disorders. Longitudinal designs with repeated measures and individual RDoC trajectories would allow evaluation of sensitivity to clinical change. From a clinical perspective, our results suggest that NLP-derived RDoC scores could provide a scalable method for assessing symptom dimensions in large patient cohorts, including population-based samples within hospital systems. Application in broader and more diverse datasets will be important to establish robustness and generalizability across clinical settings.

Methodologically, newer large language models may complement existing NLP pipelines by improving the extraction of temporally and contextually qualified symptom information from clinical text. Such approaches could enable more precise reconstruction of longitudinal RDoC domain profiles while preserving compatibility with established token-based methods. Given the cost and scalability constraints associated with large language models, however, a substantial share of the relevant gains may be achievable with lighter-weight additions—most directly, a negation-detection component (e.g., NegEx/ConText) to filter negated mentions, or the targeted use of a language model only to re-adjudicate text segments in which a lexicon match has already been identified.

## Supplementary information

Supplementary Material

## Data Availability

Due to the inclusion of sensitive personal health information, individual-level data cannot be made publicly available. Summarized data that support the findings of this study are available from the corresponding author upon reasonable request.

## References

[1] GBD 2019 Diseases and Injuries Collaborators. Global burden of 369 diseases and injuries in 204 countries and territories, 1990–2019: a systematic analysis for the Global Burden of Disease Study 2019. Lancet. 2020;396:1204–22. 10.1016/S0140-6736(20)30925-9 PMC7567026 33069326

[2] World Health Organization. Depression and Other Common Mental Disorders: Global Health Estimates. 2017.

[3] Walker ER, McGee RE, Druss BG. Mortality in mental disorders and global disease burden implications: a systematic review and meta-analysis. JAMA Psychiatry. 2015;72:334–41. 10.1001/jamapsychiatry.2014.2502 PMC4461039 25671328

[4] Cuthbert BN, Insel TR. Toward the future of psychiatric diagnosis: the seven pillars of RDoC. BMC Med. 2013;11:126. 10.1186/1741-7015-11-126 PMC3653747 23672542

[5] Insel TR. The NIMH research domain criteria (RDoC) Project: precision medicine for psychiatry. Am J Psychiatry. 2014;171:395–7. 10.1176/appi.ajp.2014.14020138 24687194

[6] Insel T, Cuthbert B, Garvey M, Heinssen R, Pine DS, Quinn K, Sanislow C, Wang P. Research domain criteria (RDoC): toward a new classification framework for research on mental disorders. Am J Psychiatry. 2010;167:748–51. 10.1176/appi.ajp.2010.09091379 20595427

[7] Cuthbert BN. The RDoC framework: facilitating transition from ICD/DSM to dimensional approaches that integrate neuroscience and psychopathology. World Psychiatry Off J World Psychiatr Assoc. 2014;13:28–35. 10.1002/wps.20087 PMC3918011 24497240

[8] Der-Avakian A, Markou A. The neurobiology of anhedonia and other reward-related deficits. Trends Neurosci. 2012;35:68–77. 10.1016/j.tins.2011.11.005 PMC3253139 22177980

[9] Whitton AE, Treadway MT, Pizzagalli DA. Reward processing dysfunction in major depression, bipolar disorder and schizophrenia. Curr Opin Psychiatry. 2015;28:7–12. 10.1097/YCO.0000000000000122 PMC4277233 25415499

[10] LeDoux J, Daw ND. Surviving threats: neural circuit and computational implications of a new taxonomy of defensive behaviour. Nat Rev Neurosci. 2018;19:269–82. 10.1038/nrn.2018.22 29593300

[11] Huys QJM, Browning M, Paulus MP, Frank MJ. Advances in the computational understanding of mental illness. Neuropsychopharmacology. 2021;46:3–19. 10.1038/s41386-020-0746-4 PMC7688938 32620005

[12] Fernandes BS, Williams LM, Steiner J, Leboyer M, Carvalho AF, Berk M. The new field of ‘precision psychiatry’. BMC Med. 2017;15:80. 10.1186/s12916-017-0849-x PMC5390384 28403846

[13] Torous J, Baker JT. Why psychiatry needs data science and data science needs psychiatry: connecting with technology. JAMA Psychiatry. 2016;73:3–4. 10.1001/jamapsychiatry.2015.2622 26676879

[14] McCoy TH Jr, Yu S, Hart KL, Castro VM, Brown HE, Rosenquist JN, Doyle AE, Vuijk PJ, Cai T, Perlis RH. High throughput phenotyping for dimensional psychopathology in electronic health records. Biol Psychiatry. 2018;83:997–1004. 10.1016/j.biopsych.2018.01.011 PMC5972065 29496195

[15] McCoy TH, Pellegrini AM, Perlis RH. Research domain criteria scores estimated through natural language processing are associated with risk for suicide and accidental death. Depress Anxiety. 2019;36:392–9. 10.1002/da.22882 PMC6488379 30710497

[16] McCoy TH Jr, Han L, Pellegrini AM, Tanzi RE, Berretta S, Perlis RH. Stratifying risk for dementia onset using large‐scale electronic health record data: a retrospective cohort study. Alzheimers Dement. 2020;16:531–40. 10.1016/j.jalz.2019.09.084 PMC7067642 31859230

[17] McCoy TH, Wiste AK, Doyle AE, Pellegrini AM, Perlis RH. Association between child psychiatric emergency room outcomes and dimensions of psychopathology. Gen Hosp Psychiatry. 2019;59:1–6. 10.1016/j.genhosppsych.2019.04.009 PMC7392621 31034963

[18] Castro VM, Gunning FM, McCoy TH, Perlis RH. Mood disorders and outcomes of COVID-19 hospitalizations. Am J Psychiatry. 2021;178:541–7. 10.1176/appi.ajp.2020.20060842 PMC11905964 33820425

[19] McCoy, T CQH-Dimensional-Phenotyper, https://github.com/thmccoy/CQH-Dimensional-Phenotyper.

[20] Rush AJ, Trivedi MH, Ibrahim HM, Carmody TJ, Arnow B, Klein DN, Markowitz JC, Ninan PT, Kornstein S, Manber R, Thase ME, Kocsis JH, Keller MB. The 16-Item quick inventory of depressive symptomatology (QIDS), clinician rating (QIDS-C), and self-report (QIDS-SR): a psychometric evaluation in patients with chronic major depression. Biol Psychiatry. 2003;54:573–83. 10.1016/s0006-3223(02)01866-8 12946886

[21] Snaith RP, Hamilton M, Morley S, Humayan A, Hargreaves D, Trigwell P. A scale for the assessment of hedonic tone the snaith-hamilton pleasure scale. Br J Psychiatry J Ment Sci. 1995;167:99–103. 10.1192/bjp.167.1.99 7551619

[22] Spitzer RL, Kroenke K, Williams JBW, Löwe B. A brief measure for assessing generalized anxiety disorder: the GAD-7. Arch Intern Med. 2006;166:1092–7. 10.1001/archinte.166.10.1092 16717171

[23] Khazanov GK, Ruscio AM, Forbes CN. The positive valence systems scale: development and validation. Assessment. 2020;27:1045–69. 10.1177/1073191119869836 31416336

[24] R Core Team. R: A Language and Environment for Statistical Computing. (R Foundation for Statistical Computing, 2024). https://www.R-project.org/.

[25] Revelle, W psych: Procedures for Psychological, Psychometric, and Personality Research. 2.5.6 2007. 10.32614/CRAN.package.psych.

[26] Romer AL, Bertocci MA, Phillips ML, Pizzagalli DA. Neuroimaging and Circuit Mechanisms of Depression. in Charney and Nestler's Neurobiology of Mental Illness (eds. Charney DS, Nestler EJ, Buxbaum JD, Binder EB, Gordon JA, Picciotto MR) 375–392 (Oxford University Press, 2025). 10.1093/med/9780197640654.003.0029.

[27] Pizzagalli DA. Depression, stress, and anhedonia: toward a synthesis and integrated model. Annu Rev Clin Psychol. 2014;10:393–423. 10.1146/annurev-clinpsy-050212-185606 PMC3972338 24471371

[28] Pizzagalli DA, Roberts AC. Prefrontal cortex and depression. Neuropsychopharmacol Off Publ Am Coll Neuropsychopharmacol. 2022;47:225–46. 10.1038/s41386-021-01101-7 PMC8617037 34341498

[29] Williams LM. Precision psychiatry: a neural circuit taxonomy for depression and anxiety. Lancet Psychiatry. 2016;3:472–80. 10.1016/S2215-0366(15)00579-9 PMC4922884 27150382

[30] Etkin A, Wager TD. Functional neuroimaging of anxiety: a meta-analysis of emotional processing in PTSD, social anxiety disorder, and specific phobia. Am J Psychiatry. 2007;164:1476–88. 10.1176/appi.ajp.2007.07030504 PMC3318959 17898336

[31] McLean CP, Levy HC, Miller ML, Tolin DF. Exposure therapy for PTSD: a meta-analysis. Clin Psychol Rev. 2022;91:102115. 10.1016/j.cpr.2021.102115 34954460

