# Supplementary material for: Alignment of natural language processing-derived RDoC scores with depression, anhedonia, and anxiety in psychiatric electronic health records
Source: NPP Digit Psychiatry Neurosci. 2026 Sep 30;4:19. doi: 10.1038/s44277-026-00070-3 (PMC13627714; doi:10.1038/s44277-026-00070-3)
Supplement: Supplementary file 1 [file 44277_2026_70_MOESM1_ESM.pdf]

Supplementary materials

Supplementary Table 1: Model fit statistics for multivariable regression analyses of psychometric assessments.

Supplementary Figure 1: Univariable comparisons between questionnaire completers and non-completers.

Supplementary Figure 2: Multivariable predictors of inclusion in each questionnaire analytic sample.

Supplementary Figure 3: Pairwise lexical overlap between RDoC domain lexicons (Jaccard index).

**Assessment**	N	R^2	Adjusted R^2
PVSS (Positive Valence)	323	0.04	0.02
QIDS (Depression)	101	0.18	0.12
SHAPS (Anhedonia)	127	0.11	0.05
GAD-7 (Anxiety)	153	0.06	0.02

Supplementary Table 1: R^2 represents the proportion of variance in each psychometric assessment explained by the regression model; adjusted R^2 provides a corrected estimate that accounts for the number of predictors and sample size.

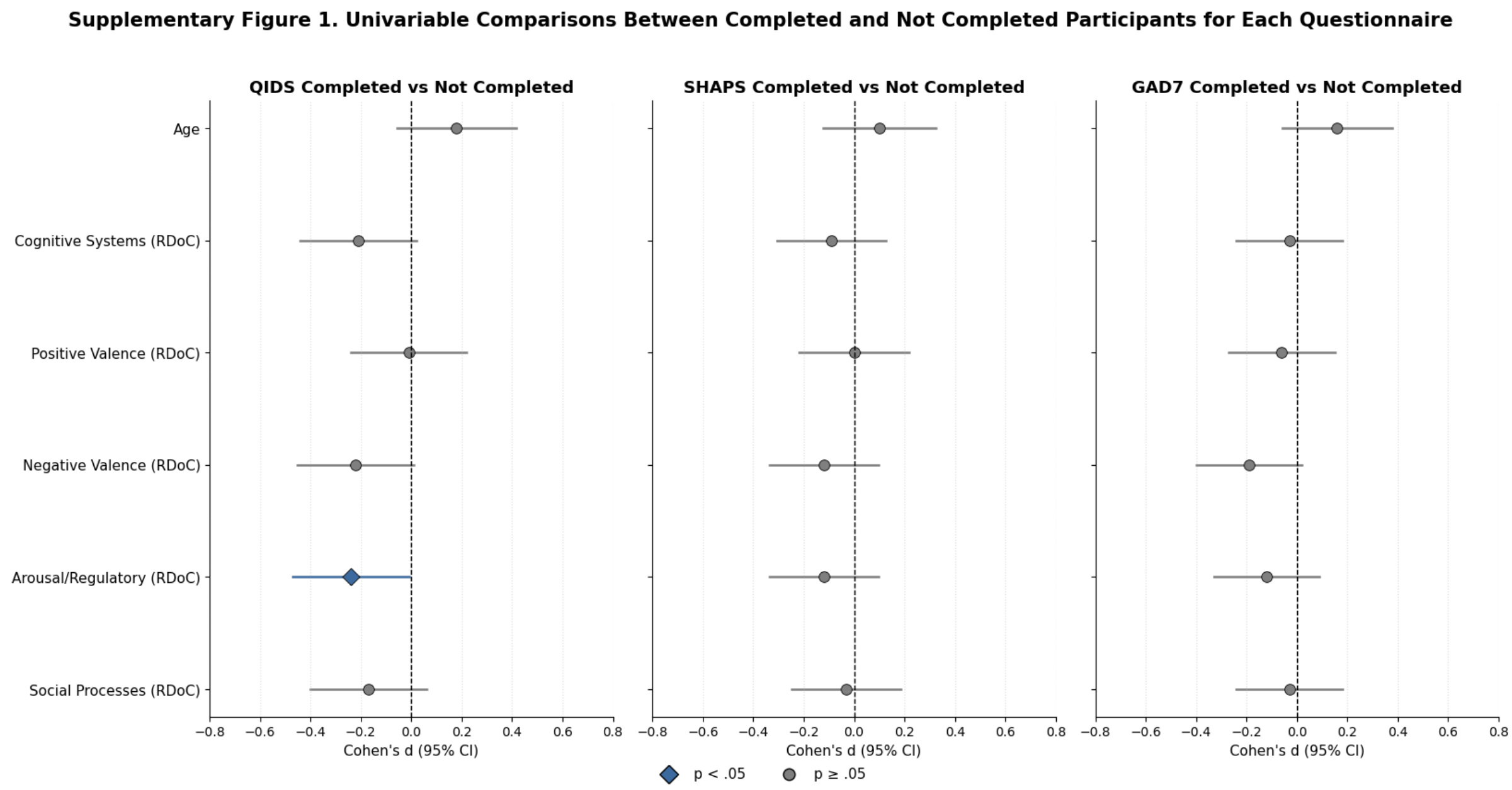

Supplementary Figure 1: Univariable comparisons of demographic characteristics and NLP-derived RDoC domain scores between questionnaire completers and non-completers. Points represent Cohen's d and horizontal bars indicate 95% confidence intervals. Blue diamond denotes comparisons with p < .05. PVSS was not included because all participants completed this questionnaire; exclusions from the PVSS analytic sample were due only to missing covariate data in the complete-case regression analyses.

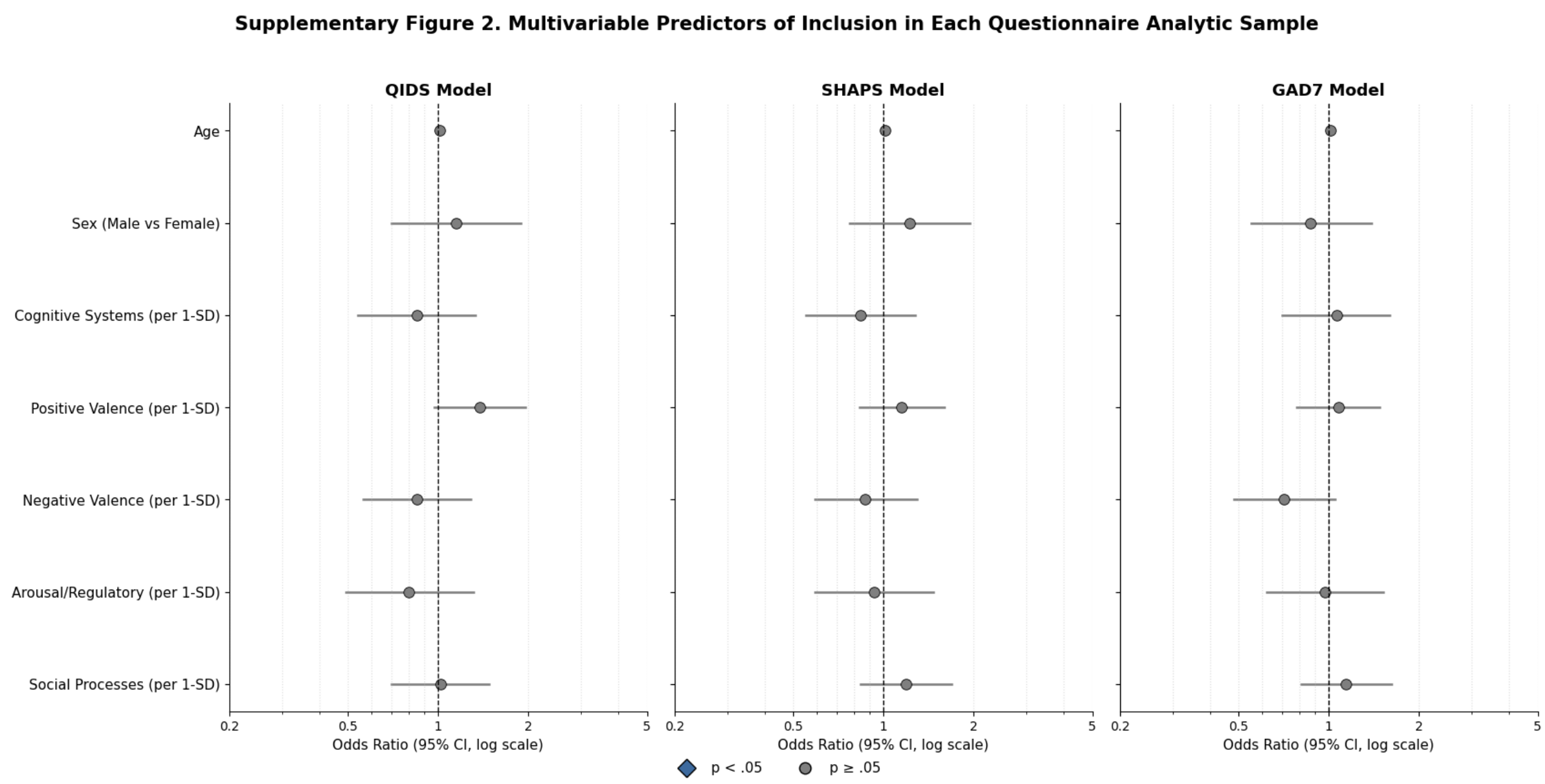

Supplementary Figure 2: Multivariable logistic regression predicting inclusion in each questionnaire analytic sample. PVSS was not included because inclusion in the analytic sample depended only on complete covariate information rather than questionnaire completion.

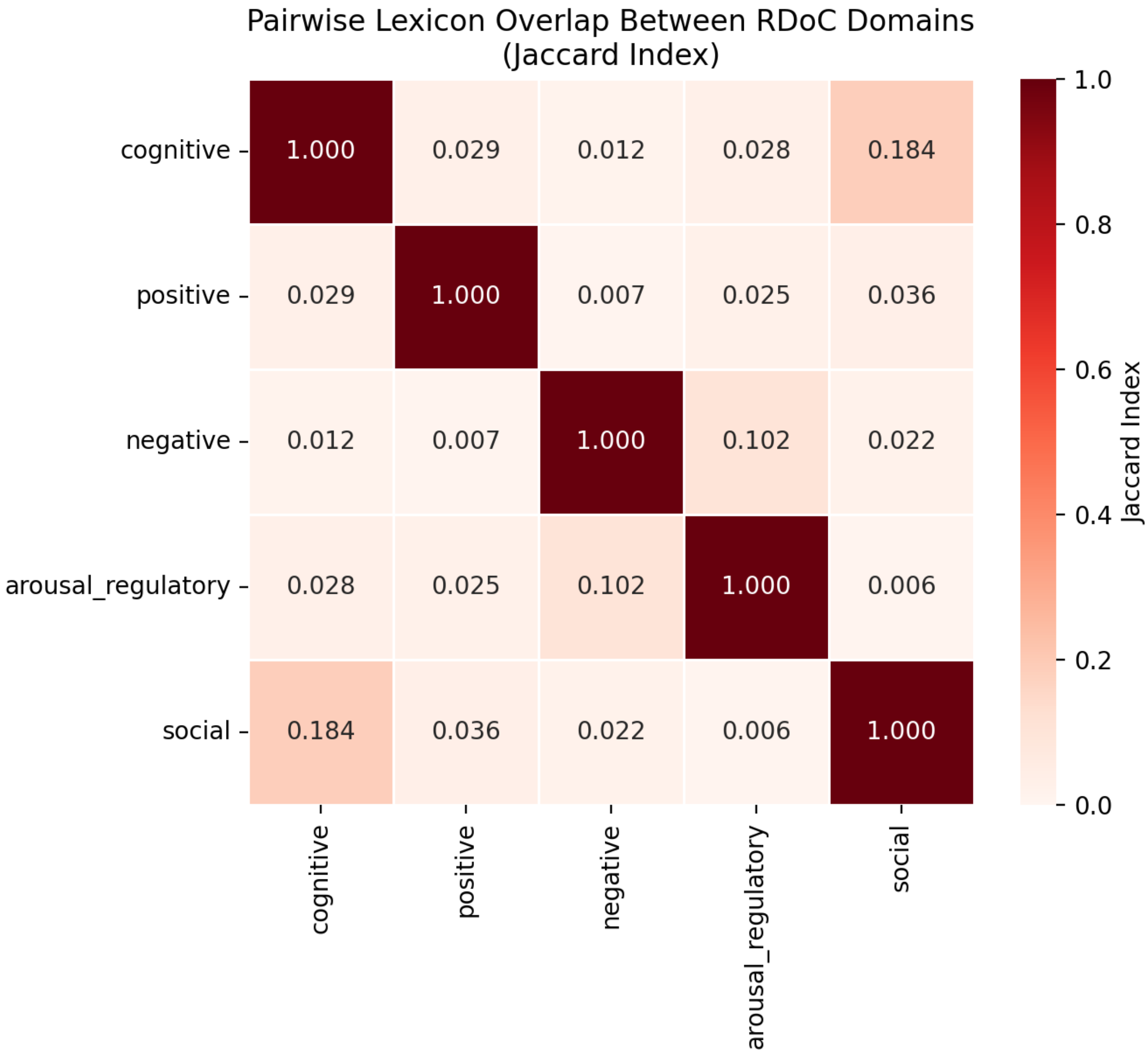

Supplementary Figure 3: Pairwise lexical overlap between RDoC domain lexicons (Jaccard index). Cells indicate pairwise Jaccard similarity coefficients between the lexicons defining each RDoC domain. Higher values indicate greater lexical overlap.
